# Depression, Anxiety and Stress Among Women Treated Due to Infertility

**DOI:** 10.3390/jcm15135086

**Published:** 2026-06-30

**Authors:** Kamila Wójtowicz, Justyna Kot, Marta Makara-Studzińska, Natalia Wdowiak, Melania Bojar, Andrzej Wróbel, Dominika Trojnarska, Artur Wdowiak

**Affiliations:** 1 Gynecology and Obstetrics Department, Medical Center in Łańcut, 37-100 Łańcut, Poland; guccii@poczta.onet.pl; 2 Department of Maternal and Child Health, Institute of Nursing and Midwifery, Faculty of Health Sciences, Jagiellonian University Medical College, Kopernika 25 Street, 31-501 Kraków, Poland; dominika.trojnarska@uj.edu.pl; 3 Faculty of Health Sciences, Jagiellonian University Medical College, Kopernika 25 Street, 31-501 Kraków, Poland; mmakarastudzinska@gmail.com; 4 Beautiful Smile Clinic, Narutowicza 68 Street, 20-013 Lublin, Poland; natalia.wdowiak62@gmail.com; 5 Department of Obstetrics and Gynecology, Faculty of Health Sciences, Medical University of Lublin, Staszica 4-6 Street, 20-081 Lublin, Poland; melabojar@gmail.com (M.B.); wdowiakartur@gmail.com (A.W.); 6 Second Department of Gynecology, Medical University of Lublin, Jaczewskiego 8 Street, 20-090 Lublin, Poland; wrobelandrzej@yahoo.com

**Keywords:** depression, anxiety, stress, infertility, reproductive techniques

## Abstract

**Background/Objectives**: Infertility is an important public health problem. It is the source of considerable psychological load, negatively affecting the quality of life, social relations, and psychological health. Stress in infertility plays the role of both a risk factor and a consequence of reproductive disorders, exerting an effect through hormonal, inflammatory and oxidative mechanisms, which may reduce the effectiveness of treatment. The aim of the study is assessment of the frequency of occurrence of depression, anxiety, and stress in infertile women. **Methods**: The study included 300 women treated due to infertility, and 50 women in the control group. The evaluation was carried out using an author-constructed socio-demographic questionnaire and the Depression Anxiety Stress Scales-42 (DASS-42) for the measurement of depression, anxiety, and stress. In addition, selected hormones were determined in blood serum. **Results**: Hormonal analysis showed significantly higher levels of prolactin (PRL) in women treated due to infertility, especially in the group without the use of assisted reproductive technology (non-ART), whereas higher levels of the follicle-stimulating hormone (FSH), and luteinizing hormone (LH) were noted in the control group. The level of anti-mullerian hormone (AMH) was lower in the therapeutic group, with no differences observed between the methods of treatment, and the levels of thyroid-stimulating hormone (TSH) did not differ between groups. Psychological evaluation according to the DASS-42 showed a significantly higher intensity of depression, anxiety, and stress in patients treated due to infertility, this intensity being the highest in the in vitro fertilization (IVF) group. Weak, but significant, correlations were found between PRL, FSH and LH, and the intensity of the symptoms of depression, anxiety, and stress, and the overall result according to the DASS-42. **Conclusions**: Infertile women experienced higher levels of depression, anxiety, and stress, compared to fertile women. The study suggests a relationship between the level of psychological stress in persons treated for infertility, and their hormonal status. The study demonstrates that integration of psychological support and infertility treatment may bring about important benefits for psychological health and the quality of life of patients.

## 1. Introduction

The report presented by the World Health Organization (WHO) estimates that one in six people globally are affected by infertility, i.e., even 17.5% of the population [1]. Infertility is a situation in which a couple cannot achieve a clinical pregnancy, with regular sexual intercourse (two to four times a week) for a period of minimum 12 months, without using any contraceptive methods. The main consequence of infertility is childlessness, which contributes to the deterioration of the quality of life from many aspects. Simultaneously, this problem exerts a negative effect on psychological health, leading to higher levels of stress, which may additionally have a negative impact on conceiving a child [2,3].

Poland is currently experiencing a sustained negative demographic trend. Although this phenomenon is multifactorial, reproductive health problems may constitute a significant contributing factor. The most recent assessment of infertility prevalence among couples in Poland was provided by the 2024 National Fertility Survey, the findings of which suggest an escalating reproductive health challenge. The study highlights insufficient health awareness as a key issue and indicates that a considerable proportion of respondents—approximately one in five (25% of women and 12% of men)—report experiencing fertility-related difficulties. This corresponds to an estimated total of up to 1.5 million affected couples nationwide [4,5,6].

The physiological dimension of stress is very complex. Stress may be both the effect and the risk factor, as it exerts a biological effect by affecting the release of hormones. On the one hand, studies show a positive correlation between the level of cortisol (the stress hormone) in blood, and the intensity of anxiety and depression in patients treated due to infertility. On the other hand, stress and the released substances may impair reproductive functions and determine the effectiveness of treatment. Stress related with therapy may, to a great extent, affect the physiology of the body. Its chronic effect causes an increase in oxidative stress and inflammatory factors. This, in turn, may contribute to the problem with achieving a clinical pregnancy [7,8].

Inability to conceive affects social bonds, including relationships between partners. Among both males and females treated for infertility considerable lowering of mood, decreased self-esteem, helplessness, and despondency are observed. Stress related to pressure and critical evaluation by the environment intensify negative emotions. Infertility constitutes a serious emotional challenge, especially for women. Studies suggest that the female gender is more predisposed to the development of psychological disorders resulting from the pressure of conceiving a child. Infertile women more often declare the feeling of frustration, disappointment and guilt. They also frequently experience an intense physical fatigue, loss of sense of attractiveness, and sexual dysfunctions [3,9,10,11,12].

Many studies indicate a significant correlation between the quality of life and the level of psychological stress in infertile women. As is well known, high levels of stress, anxiety, and depression may exert a negative effect on the quality of life of such patients, compared to women with confirmed fertility. In addition, it was observed that the procedures related to assisted reproductive technology (ART) also suggest a considerable stress [13,14].

Among the tools for the assessment of the psychological status among the population undergoing infertility treatment are the Depression Anxiety Stress Scales (DASS), for the measurement of depression, anxiety and stress. The DASS are internationally recognized and well-verified instruments for the measurement of negative emotional states related with depression, anxiety, and stress, in both the healthy and sick populations. There are several adaptations of the DASS, such as the version for adolescents (the Depression Anxiety Stress Scales-Youth version, DASS-Y), or abbreviated forms (Mini-DASS). The DASS-42 and DASS-21 are the most popular and most frequently used versions. Analyses of data using this measure provided consistent results concerning its psychometric properties. The DASS questionnaire has found broad application in scientific research conducted in many countries [15,16,17].

A substantial body of the literature has documented increased levels of depression, anxiety, and stress among women experiencing infertility; however, the association between psychological distress and hormonal status remains insufficiently elucidated. In particular, there is a paucity of evidence regarding potential differences in psychological burden across various infertility treatment modalities, including non-assisted approaches (non-ART), intrauterine insemination (IUI), and in vitro fertilization (IVF). Furthermore, it remains unclear whether the type of treatment is linked to differential emotional responses and endocrine profiles. Therefore, the present study seeks to address this gap by concurrently assessing psychological distress and selected hormonal parameters in women undergoing diverse infertility treatment strategies.

The aim of the present study was assessment of the frequency of occurrence of depression, anxiety, and stress in infertile women.

## 2. Materials and Methods

### 2.1. Study Groups

The presented study was conducted during the period from 2023 to 2025, after obtaining the approval of the Bioethics Committee at the Medical University in Lublin. The study group included 300 respondents divided into appropriate subgroups, according to the implemented therapeutic procedure, i.e., 100 patients in the non-ART, intrauterine insemination (IUI), and in vitro fertilization (IVF) group, each. In turn, the control group included 50 women with confirmed fertility. A questionnaire containing socio-demographic data and the DASS-42 was completed directly by respondents in the study group before treatment. The absence of psychological disorders was based solely on participants’ self-report. Participants were recruited using two complementary approaches. In the study group, women undergoing infertility treatment were invited to participate during visits at an infertility clinic; recruitment was based on voluntary participation and was not strictly consecutive. In the control group, participants were recruited through an online invitation. Announcement was posted via online platforms, and individuals who responded subsequently presented at the clinic, where the recruitment process was continued. The respondents were qualified into the study based on the following criteria:

- Study group—women qualified for infertility treatment who expressed their consent to participate in the study, without psychological disorders;
- Control group—women with confirmed fertility who expressed their consent to participate in the study, without psychological disorders.

The criteria of exclusion from the study and control groups were as follows:

- Lack of consent to participate in research, treatment due to psychological disorders, ovulatory disorders diagnosed in accordance with the WHO criteria.

**Figure 1:**
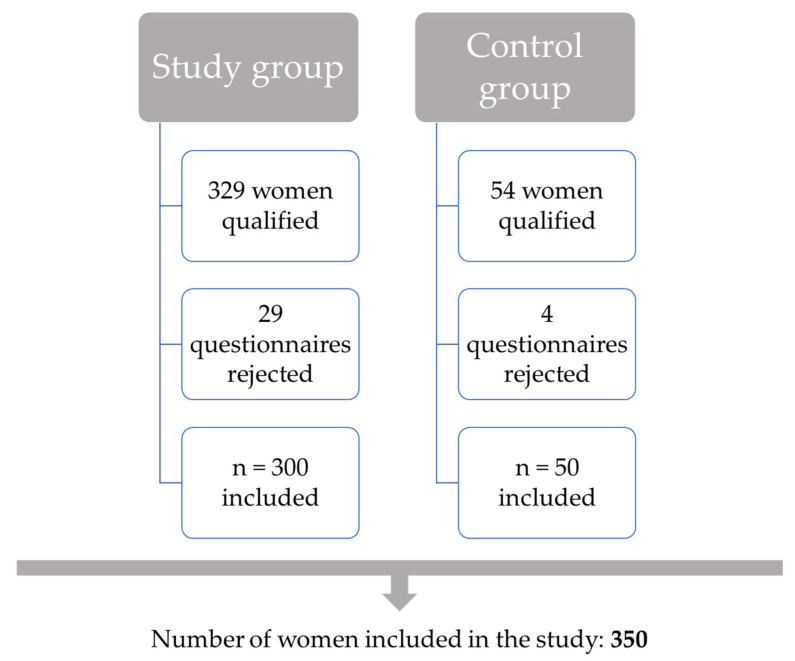
Sample selection flowchart.

After analysis of the collected material, 33 questionnaires were excluded from the study due to the insufficient amount of data, which made impossible the complete analysis of cases (29 in the study group and 4 in the control group; respectively). Figure 1 presents a flowchart.

### 2.2. Measurement Tools

During the study the women were asked to complete an author-constructed socio-demographic questionnaire, and the Polish version of the validated DASS-42 questionnaire. The scale of depression, anxiety, and stress is a self-report questionnaire for the assessment of the risk of occurrence of the symptoms of emotional disorders. The examined woman evaluated how much the statement applied to her in the past week, using a 4-point intensity/frequency scale. Each of the DASS contains 14 items. The scale of depression assesses dysphoria, hopelessness, devaluation, lack of interest/engagement, anhedonia and apathy. The scale of anxiety takes into account autonomic arousal, skeletal muscle tension, situational anxiety and the subjective experience of anxiety affect. The assessed group of symptoms of stress includes difficulty relaxing, nervous agitation, easily irritated, irritability/overreaction, and impatience. The results for depression, anxiety, and stress are calculated by summing up the results for the appropriate items. It should be emphasized that this scale is based on a dimensional, not a categorical, understanding of the disorder. Thus, the DASS-42 is not a direct diagnostic tool (does not make a psychiatric diagnosis), but helps to identify areas requiring specialist’s attention. This tool may be successfully applied in both healthy and clinical individuals [18].

The levels of the follicle-stimulating hormone (FSH), luteinizing hormone (LH), prolactin (PRL), and anti-mullerian hormone (AMH) were measured on the third day of the cycle preceding ovulation. The levels of all hormones, including thyroid-stimulating hormone (TSH), were determined in serum collected from morning blood samples (5 mL).

### 2.3. Statistical Analysis

Statistical analyses were conducted using STATISTICA version 13.3 software. Before selecting the appropriate statistical tests, their assumptions were verified. Continuous variables were assessed for normality using the Shapiro–Wilk test. Student’s *t*–test was used to compare quantitative variables between groups with distributions close to normal. F test of analysis of variance was used to compare numerical variables between study groups. Lindenberg–Levy central limit theorem was used for an arithmetic mean in a large sample. Frequency analysis and the Pearson’s chi-square (χ^2^) test were used to check if there was an association between qualitative data. Pearson correlation coefficient (*r*) was used to correlate DASS scores with hormonal status in women treated for infertility. The significance level was assumed to be α = 0.05 for all analyses. Statistically significant results are presented in bold font. If differences between groups or correlations were significant, we also assessed effect size using Cohen’s d for Student’s test for 2 means in 2 unpaired samples, eta-squared (η^2^) for F test of analysis of variance, and Pearson correlation coefficient with values that were at least 0.3.

Sample size calculation, conducted for F test of analysis of variance to compare the total score of the DASS between four groups of women, with 80% power, at a significance level of 0.05, concluded that 50 is enough for each group.

## 3. Results

The study group included 300 patients treated due to infertility, whereas the control group included 50 women. The mean age of respondents in the study group was within the range 32–34, while in the control group it was 34 years (statistically significant relationship; *p* = 0.020). According to the level of education, the vast majority of respondents had higher education. With respect to the place of residence, the largest number of respondents were inhabitants of large cities. According to the BMI, the group treated for infertility most often represented normal body weight, similar to the control group. In the context of time devoted to trying to have a child, irrespective of the method applied, this period was approximately 3–3.5 years, on average. Analysis of answers to the question concerning the possession of a child indicated that women treated by the non-ART method more often had offspring, compared to patients from the IUI and IVF groups (34% vs. 18%; *p* < 0.001). The majority of respondents from both groups performed non-manual type of work. Among the respondents the largest number of women had a net income per household member exceeding 2000 PLN per month. Table 1 presents the characteristics of the study group.

Analysis of the respondents’ hormonal status (Table 2 and Table 3) demonstrated that higher values of the PRL level were found in the group treated due to infertility, of which the highest result represented women undergoing treatment using the non-ART method (*p* < 0.001, compared to the control and IUI groups, as well as *p* = 0.001 vs. IVF).

Higher FSH and LH values were characteristic of the control group (*p* < 0.001), whereas in the therapeutic group individual hormones reached similar levels.

The level of AMH was statistically significantly lower values among women treated due to infertility compared to control group (*p* < 0.001). No differences were observed within the group undergoing therapy. In turn, no differences in the TSH were observed between the control and the study groups.

Table 4 presents the results according to the DASS. The measurement showed that the group of healthy women, compared to patients treated due to infertility, obtained significantly lower results according to all three scales, as well as the overall result (*p* < 0.001). In the group of women undergoing therapy it was observed that the assessed psychological parameters, i.e., depression, anxiety, and stress, reached the highest values in the case of IVF treatment. Evaluation of within-group differences also indicated a significant increase in symptoms in the IUI group, compared to the patients treated by the non-ART techniques.

Table 5 presents correlations between the hormonal status and the results according to three scales, and the overall result according to the DASS-42. No statistically significant relationship was found between the hormonal status and the subscale *Anxiety*. It was noted that the levels of AMH and TSH did not correlate with the results of individual scales, and the overall DASS score.

Based on the results obtained it may be presumed that the levels of PRL, FSH and LH were significantly related with the subscales *Depression*, *Stress* and the *Total score* result. Correlation analysis showed a positive relationship among an increase in PRL, the intensification of the symptoms of depression and stress, and the overall result (*p* < 0.001). It may be observed that lower levels of FSH and LH were related with higher values of the intensity of the symptoms of depression and anxiety, and the *Total score* results obtained according to the DASS questionnaire (*p* < 0.001). The strength of these relationships was weak according to the correlation coefficient which was ±0.2 ÷ 0.3.

## 4. Discussion

Difficulties with reproduction and fertility are a serious problem, which extends far beyond the medical dimension. In the presented study, an attempt was undertaken to assess the frequency of occurrence of depression, anxiety, and stress in women diagnosed with the problem of infertility. The study was conducted using the diagnostic method in the form of the Polish adaptation of the Depression Anxiety Stress Scale (DASS). To the best of our knowledge, this scale is a reliable and sensitive tool for the assessment of the psychological status, which may be effectively applied in the case of women with infertility [18].

As is well known, various factors, such as age, duration of infertility, cause of infertility, treatment history, level of education, occupational status, income, and pressure from the environment, may contribute to stress in women experiencing infertility. In this study, individual respondents’ socio-demographic characteristics were relatively homogeneous. Among these data, a statistically significant relationship was observed only with respect to age. The results obtained by Park indicate that despite the fact that the indices of the prevalence of anxiety, depression, and stress were higher in women experiencing infertility than in the control group, they did not correlate with age and duration of infertility [19]. As can be observed in the present study women treated by the IUI and IVF methods more often suffered from primary infertility, compared to the patients undergoing non-ART treatment.

Considering the results according to the DASS it was confirmed that women treated due to infertility presented higher values in the subscales of depression, anxiety and stress, and in the overall result. An increase in the symptoms was particularly clear in the group receiving treatment by the IVF method, followed by the IUI and non-ART methods.

Available evidence indicates that psychological distress remains elevated among patients undergoing both IUI and IVF, with no consistent association with procedural invasiveness. In the context of in vitro fertilization, stress levels demonstrate temporal variability across treatment stages (e.g., ovarian stimulation, embryo transfer), underscoring the role of situational determinants. This relationship is inherently complex and influenced by multiple confounding variables, including clinical factors (duration of infertility, number of prior treatment attempts, prognosis), psychosocial conditions (availability of support, socio-cultural expectations), and individual patient characteristics, rather than being attributable solely to the type of assisted reproductive technique employed [20,21,22].

The results of other studies clearly indicate that infertility is accompanied by depression, anxiety, and stress. A study conducted in South Korea among infertile women showed higher levels of psychological stress measured by means of the DASS-42, compared to fertile women. Similar results were obtained in Pakistan, where infertile women had higher results regarding depression, anxiety, and stress, compared to the control group. Also, another study conducted in India demonstrated that the majority of infertile women experienced serious emotional stress. The results obtained by Chi et al. and Yusuf showed that the mean scores according to individual subscales were lower than in the present study. In turn, the mean DASS–42 total score obtained by Yadav et al. was 78 [9,23,24]. The differences obtained in the levels of depression, anxiety, and stress related with infertility may result from the cultural context.

Nearly half of the Polish respondents in the National Fertility Test reported that they would not consider in vitro fertilization for reasons other than financial constraints. In this context, non-financial barriers to IVF predominantly encompass ethical, religious, and socio-cultural considerations, as well as health-related concerns, insufficient knowledge, and psychological factors. Collectively, these determinants may substantially increase the psychological burden experienced by patients, contributing to elevated levels of stress and anxiety. Moreover, perceived social pressure and uncertainty regarding the course and outcomes of the procedure may further exacerbate psychological distress and reduce patients’ sense of control over the treatment process [5,25].

Cultural differences exert an effect on the intensity of symptoms. A systematic review and meta-analysis by Simbar et al. indicated a disproportion in the level of infertility-related stress between developed and developing countries. In countries with a high economic and social development index broad access to health care is widespread, which translates into better access to medical care, and the treatment of infertility is also more frequent. Patients in these countries receive greater support and are less exposed to social stigma, which means a lower level of stress. In turn, in developing economies, access to health care, diagnosis, and therapy is very limited. Couples struggling with infertility, especially women, frequently experience ostracism, stigmatization, and social pressure. As a result, inhabitants of low- and middle-income countries experience greater stress related with infertility, which is intensified by cultural and social norms which highly value having children. Similar observations are cited by Chaurasiya et al. According to this study, in the Indian socio-cultural context, prolonged infertility or repeated miscarriages often expose women to constant family pressure, social comparisons, and stigmatization, which may intensify stress [26,27].

The results of the present study regarding hormonal balance demonstrated that the PRL serum concentration was higher and AMH concentration was lower in the group undergoing treatment. In turn, the levels of FSH and LH were higher in the control group. No differences in the level of TSH were found between the treated and the control groups.

One possible explanation for the lower FSH and LH levels observed in the study group is the higher prevalence of subclinical hypogonadotropic hypogonadism among women undergoing infertility treatment. This condition is characterized by a functionally suppressed hypothalamic–pituitary–ovarian axis that has not yet progressed to overt clinical failure. In its latent form, hormone levels may remain within the lower limits of normal, and menstruation may still occur, although cycles are often irregular or anovulatory. In these patients, impaired oocyte maturation as well as deficiencies of estradiol and progesterone during the luteal phase of the menstrual cycle may be expected, which may reduce the likelihood of achieving pregnancy. The main contributing factors include chronic stress, in which elevated cortisol levels directly inhibit hypothalamic GnRH secretion; low energy availability resulting from restrictive diets, rapid weight loss, or low body mass (i.e., reduced adipose tissue responsible for leptin production—the hormone signaling energy reserves to the brain); as well as latent hyperprolactinemia, where elevated prolactin levels (e.g., occurring only during stress or nocturnally) suppress the pulsatile secretion of LH and FSH. In the latter case, this mechanism may explain the higher prolactin (PRL) levels observed in the study group compared with the control group [28].

A literature review by Iancu et al. notes that an increased level of PRL, especially hyperprolactinaemia is the cause of infertility due to the suppressive effect on gonadotropin production. Biologically, the activity of prolactin (PRL) influences the inhibition of ovulation and menstrual cycle disturbances. In turn, during the ART treatment, especially during IVF, fluctuations in prolactin levels are possible, characterized as transient hyperprolactinaemia, the possible causes of which are administration of gonadotropins and GnRH agonists, elevated estradiol levels, and stress related to the procedure [29]. A systematic review by Karunyam et al. indicates the role of cortisol, the main stress hormone. The researchers emphasize the effect of high levels of cortisol on human reproductive function by inhibiting the release of LH and FSH. This may result in disturbed ovulation and, consequently, infertility. Simultaneously, this study provides information that although the majority of the available results of research link chronically elevated cortisol levels to impaired reproductive endocrinology, proving a direct cause–effect relationship between cortisol and fertility is difficult and requires further studies [7].

The present study concerning the hormonal profile in women treated due to infertility showed that the levels of AMH and TSH did not correlate with the results of individual subscales and the overall DASS score. The levels of PRL, FSH and LH were significantly related with the subscales Depression, Stress and the Total score. Analysis of correlations showed a positive relationship among an increase in PRL the intensity of the symptoms of depression and stress, and the overall result. However, lower FSH and LH levels were related with higher values of the intensity of depressive and stress symptoms. The results obtained differ, among others, from those by Rahimli Ocakoglu et al. According to these researchers there is no significant relationship between the levels of hormones, including AMH, TSH, FSH and PRL, and depression in infertile patients [30].

The relationship between hormonal disorders and the occurrence of the symptoms of depression and anxiety does exist. Hormones play an important role in the regulation of body functions, and also exert an effect on the mood of an individual. Disturbances in hormonal homeostasis may result in the development of depression. In this mechanism, a special role is assigned to sex hormones, the thyroid and the hypothalamic–pituitary–adrenal (HPA) axis. Hyperactivity of the HPA axis is closely related to the pathophysiology of anxiety, depression, and cognitive functioning [31].

According to the reviews by Liang et al. and Huluba et al., depressive disorders are more common in women at reproductive age, compared to men [31,32]. Kundakovic et al. point to the fluctuations in female sex hormones as the main biological factor causing sex-based differences in the risk of anxiety and depression. Estrogen and progesterone act as modulators of other neurotransmitters, one of which is the serotoninergic system. Disturbances in the secretion of sex hormones may cause potential neuropsychological dysfunctions [33]. Lower results in neuropsychological tests may also be achieved by patients with hyperprolactinaemia. Thyroid function disorders may also co-occur with the exacerbation of depressive symptoms. Thyroid hormones exert an effect on the metabolism of serotonin and noradrenaline, i.e., neurotransmitters essential in mood regulation. A cross-sectional study by Roa Duenas et al. confirmed that low levels of TSH and fluctuations of FT4 were associated with intensification of the symptoms of depression, whereby hypothyroidism was associated with fewer depressive episodes, while hyperthyroidism with increased symptoms [34].

There is a need for further studies in order to more precisely determine the interactions taking place between hormonal disorders, and the occurrence of the states of anxiety and depression. Similar to the study by Simbar et al. the results of the presented study also indicate the need for the routine psychosocial assessment in infertility [26]. In consequence, only an integrated approach combining biochemical examinations and psychological assessment may provide more precise information concerning mechanisms underlying psychological suffering in this group of patients. The role of psychological support in the course of the therapeutic process is also crucial, as well as an access to safe, effective and evidence-based infertility care.

The present study has several important advantages which prove its value and scientific, as well as practical, significance. First of all, it is worth emphasizing that it addresses an important and current problem. The scope of the study falls in an important stream of interdisciplinary research, considering the medical, psychological, and social perspectives. The results underscore the importance of integrating psychological support into infertility care and highlight the need for the introduction of interdisciplinary psychological support within infertility treatment clinics. The strengths of the study include the use of a standardized measurement tool, which increased the reliability and validity of the results obtained. Another advantage was that respondents were recruited not only in person, but also by the internet, which allowed us to expand the scope of the research. However due to the use of both clinic-based and online recruitment strategies, the response rate could not be precisely calculated, and potential differences between recruitment sources cannot be excluded. A limitation of the present study, particularly in the context of hormonal assessment, was the lack of feasibility to conduct a comprehensive diagnostic evaluation of latent hyperprolactinemia. Furthermore, the observed correlations between hormonal parameters and DASS scores were relatively weak; therefore, their clinical relevance should be interpreted with caution. The potential influence of other confounding factors that may have affected the observed associations should also be considered, including age, BMI, and duration of infertility. An additional limitation of the present study is that the absence of psychological disorders was determined exclusively on the basis of self-reported data and was not confirmed through an independent clinical assessment. Consequently, the inclusion of participants with undiagnosed or unreported psychological conditions cannot be excluded, which may have affected the DASS-42 outcomes. At the same time, it should be emphasized that the sample size, especially the control group, was relatively small, which limits the possibility of intergroup comparisons and constrains the generalizability of the results to a larger population. In addition, due to the cross-sectional nature of the study it is not possible to draw cause–effect conclusions between the examined variables. When interpreting the obtained findings, the potential for self-report bias should be considered; however, this limitation was minimized by informing participants of the anonymity of their responses. Additionally, in future studies it is worth considering the participation of couples, and carrying out the study in a wider cultural context, which would be a valuable contribution in the development of knowledge in this area.

## 5. Conclusions

Infertility treatment has been demonstrated to adversely affect patients’ emotional well-being, contributing to elevated levels of depression, anxiety, and stress. The observed relationships suggest that greater psychological distress may be linked to alterations in hormonal parameters, including prolactin and gonadotropins; however, due to the cross-sectional design, no causal conclusions can be drawn. The application of the DASS-42 questionnaire in patients undergoing infertility treatment can be used to identify women requiring psychological support.

## Figures and Tables

**Table 1:** Characteristics of the study groups.

Variable, Parameter	Unit or Category	Control Group	Women Treated for Infertility			*p*
		*n* = 50	Non–ART *n* = 100	IUI *n* = 100	IVF *n* = 100	
Age, M ± SD	years	34.2 ± 4.3	32.6 ± 4.5	33.1 ± 4.6	34.3 ± 4.4	**0.020**
Place of residence, *n* (%)	city	24 (48.00)	46 (46.00)	42 (42.00)	44 (44.00)	0.908
	town	10 (20.00)	22 (22.00)	18 (18.00)	19 (19.00)	
	rural area	16 (32.00)	32 (32.00)	40 (40.00)	37 (37.00)	
Level of education, *n* (%)	secondary	12 (24.00)	24 (24.00)	22 (22.00)	23 (23.00)	0.763
	tertiary	38 (76.00)	76 (76.00)	78 (78.00)	77 (77.00)	
BMI, M ± SD	kg/m^2^	25.89 ± 4.86	24.53 ± 4.89	24.11 ± 5.13	24.67 ± 4.34	0.226
BMI, *n* (%)	underweight	3 (6.00)	6 (6.00)	10 (10.00)	4 (4.00)	0.302
	normal weight	27 (54.00)	56 (56.00)	54 (54.00)	62 (62.00)	
	overweight	11 (22.00)	23 (23.00)	22 (22.00)	21 (21.00)	
	obesity	9 (18.00)	14 (14.00)	14 (14.00)	13 (13.00)	
Having children, *n* (%)	yes, from the current relationship	n.a.	15 (15.00)	12 (12.00)	7 (7.00)	**<0.001**
	yes, from the previous relationship	n.a.	19 (19.00)	6 (6.00)	11 (11.00)	
	no	n.a.	66 (66.00)	82 (82.00)	82 (82.00)	
Time trying to conceive a child, M ± SD	years	n.a.	3.3 ± 2.2	3.2 ± 2.2	3.5 ± 2.1	0.772
Type of job, *n* (%)	manual	9 (18.00)	14 (14.00)	17 (17.00)	14 (14.00)	0.923
	Non-manual	27 (54.00)	53 (53.00)	53 (53.00)	52 (52.00)	
	mixed	14 (28.00)	33 (33.00)	30 (30.00)	34 (34.00)	
Monthly net income per 1 person in a household (thousand PLN), *n* (%)	below 2	11 (22.00)	27 (27.00)	28 (28.00)	21 (21.00)	0.515
	2–2.5	18 (36.00)	38 (38.00)	34 (34.00)	48 (48.00)	
	above 2.5	21 (42.00)	35 (35.00)	38 (38.00)	31 (31.00)	

Student’s *t*-test or chi-square test was used. *n*, number of respondents; M, mean; SD, standard deviation; BMI, body mass index; PLN, Polish currency; n.a., not applicable. Bold *p*-values are statistically significant (*p* < 0.05).

**Table 2:** Hormonal status in the control and study groups.

Hormone (Unit), Parameter	Control Group	Women Treated for Infertility		
	*n* = 50	Non–ART *n* = 100	IUI *n* = 100	IVF *n* = 100
PRL (ng/mL), M ± SD	13.53 ± 4.33	19.99 ± 3.58	17.37 ± 3.56	18.32 ± 3.59
FSH (mIU/mL), M ± SD	10.34 ± 2.58	5.33 ± 3.42	5.34 ± 3.47	5.32 ± 3.44
LH (mIU/mL), M ± SD	8.61 ± 2.31	4.19 ± 1.20	4.33 ± 1.27	4.11 ± 1.23
AMH (ng/mL), M ± SD	6.88 ± 0.99	5.49 ± 0.53	5.59 ± 0.52	5.46 ± 0.77
TSH (mIU/L), M ± SD	1.48 ± 1.06	1.57 ± 1.09	1.59 ± 1.07	1.56 ± 1.06

*n*, number of respondents; M, mean; SD, standard deviation.

**Table 3:** *p* for comparisons of hormones’ levels between groups.

Hormone (Unit)	Control Group vs. Non–ART	Control Group vs. IUI	Control Group vs. IVF	Non–ART vs. IUI	Non–ART vs. IVF	IUI vs. IVF
PRL (ng/mL)	**<0.001**	0.094	**0.002**	**<0.001**	**0.001**	0.062
FSH (mIU/mL)	**<0.001**	**<0.001**	**<0.001**	0.984	0.983	0.967
LH (mIU/mL)	**<0.001**	**<0.001**	**<0.001**	0.424	0.638	0.211
AMH (ng/mL)	**<0.001**	**<0.001**	**<0.001**	0.180	0.744	0.163
TSH (mIU/L)	0.958	0.957	0.914	0.896	0.948	0.842

*p* for Student’s *t*-test. Bold *p*-values are statistically significant (*p* < 0.05).

**Table 4:** DASS-42 in the control and study groups.

DASS-42, Parameter	Control Group	Women Treated for Infertility			*p*	*η* ^2^
	*n* = 50	Non-ART *n* = 100	IUI *n* = 100	IVF *n* = 100		
Depression, M ± SD	9.14 ± 6.34	16.77 ± 3.37	28.56 ± 3.73	32.78 ± 2.98	**<0.001**	**0.83**
Anxiety, M ± SD	8.54 ± 5.54	16.43 ± 3.12	28.39 ± 3.60	33.39 ± 2.79	**<0.001**	**0.86**
Stress, M ± SD	11.26 ± 6.23	17.17 ± 3.87	28.26 ± 5.57	31.55 ± 2.74	**<0.001**	**0.74**
Total score, M ± SD	28.94 ± 16.55	50.37 ± 9.59	85.21 ± 12.20	97.72 ± 7.08	**<0.001**	**0.84**

*n*, number of respondents; M, mean; SD, standard deviation; *η*^2^, effect size. *p* for F test of analysis of variance. Bold *p*-values are those statistically significant (*p* < 0.05).

**Table 5:** Correlations between DASS scores and hormonal status in women treated for infertility (*n* = 300).

Hormone (Unit)	Depression		Anxiety		Stress		Total Score	
	*r*	*p*	*r*	*p*	*r*	*p*	*r*	*p*
PRL (ng/mL)	0.302	**<0.001**	0.098	0.090	0.287	**<0.001**	0.301	**<0.001**
FSH (mIU/mL)	−0.281	**<0.001**	−0.056	0.334	−0.308	**<0.001**	−0.291	**<0.001**
LH (mIU/mL)	−0.297	**<0.001**	0.012	0.836	−0.299	**<0.001**	−0.307	**<0.001**
AMH (ng/mL)	−0.019	0.743	−0.039	0.501	−0.045	0.437	0.016	0.783
TSH (mIU/L)	0.071	0.189	0.104	0.072	−0.044	0.448	−0.028	0.629

*r*—Pearson correlation coefficient. Bold *p*-values are those statistically significant (*p* < 0.05).

## Data Availability

The datasets generated during and/or analyzed during the current study are available from the corresponding author on reasonable request.
